# Why isn't everyone an evolutionary psychologist?

**DOI:** 10.3389/fpsyg.2014.00910

**Published:** 2014-08-27

**Authors:** Darren Burke

**Affiliations:** Psychology, University of NewcastleOurimbah, NSW, Australia

**Keywords:** evolutionary psychology, evolutionary biology, comparative cognition, behavioral ecology, psychology education

## Abstract

Despite a widespread acceptance that the brain that underpins human psychology is the result of biological evolution, very few psychologists in any way incorporate an evolutionary perspective in their research or practice. There have been many attempts to convince mainstream psychology of the importance of such a perspective, mostly from those who identify with “Evolutionary Psychology,” and there has certainly been progress in that direction, but the core of psychology remains essentially unevolutionary. Here I explore a number of potential reasons for mainstream psychology continuing to ignore or resist an evolutionary approach, and suggest some ways in which those of us interested in seeing an increase in the proportion of psychologists adopting an evolutionary perspective might need to modify our tactics to increase our chances of success.

If we assume that very few highly educated people don't believe in biological evolution (which is a fairly safe assumption), then it follows that the vast majority of scientifically oriented psychologists, and psychology researchers believe that the neural mechanisms that underpin our psychological abilities and propensities are the product of evolution—of natural, kin, and sexual selection. It is puzzling, therefore, that there is not a more widespread acceptance of the importance of an evolutionarily informed approach in our science. Despite an increasing awareness and acceptance of Evolutionary Psychology (EP), it is not an exaggeration to say that almost all of the research that happens in psychology (excluding those areas explicitly interested in evolution, like EP and comparative psychology), and almost all of the applications of psychology, *completely* ignore the evolutionary origin of the mechanisms being studied, or the “principles” being applied. This is despite a series of spirited, and well-informed calls-to-arms and clarifications, each making the case that an evolutionary approach is fundamentally important, and cogently dispelling a series of prevailing myths about what such an approach entails (e.g., Barkow et al., 1992; Buss et al., 1998; Buss, 2005; Confer et al., 2010; Cosmides and Tooby, 2013). Rather than simply adding my voice to those explaining the nature and virtues of an evolutionary approach to psychology, my aim in the current essay is to suggest some ideas about why the case that has been put may not be having the traction those of us in the field had hoped it would. The perspective I am providing is that of a researcher who is involved in both EP, as it is applied to understanding human psychological mechanisms, and the ecological approach to comparative cognition, which attempts to understand how selective forces shape cognitive mechanisms in non-humans, as well as that of an academic who has taught psychology (and some biology) during the 20 years that both these research enterprises have grown in influence and popularity. My hope is to be able to highlight some potential barriers to a widespread acceptance of the centrality of evolution in psychology, and to suggest some ways in which we may be able to move forward.

While the list below is likely to be an underestimate of the factors involved, and reflects personal observations, to some extent (and so may be less true of psychology sub-disciplines I am less familiar with), I believe that there are at least six fairly straightforward explanations for continued resistance to adopting a thoroughgoing evolutionary approach in mainstream psychology, each of which will be explored in more detail. The factors are not completely independent, and no doubt interact with each other, which will complicate the picture, but hopefully by making them explicit, we will be able to better understand both the nature of the forces that need to be overcome and the weakness of the position that they represent.

1. The primacy of mechanism.
2. The identification of EP with particular versions of it.
3. Just so story telling.
4. Motivated opposition.
5. Theoretical inertia and misguided skepticism.
6. Poor understanding of modern evolutionary principles in psychology.

## The primacy of mechanism

For many psychology researchers the fact that a mechanism is the result of past evolutionary forces is assumed to be true (at least in principle), but it is also assumed to be essentially irrelevant for understanding how the mechanism works, which is the main aim of most psychological research. This perspective is frequently (and fairly) criticized for providing an incomplete understanding of the mechanism in question, since it ignores it's evolved *function*, but I think there is a danger that ignoring evolutionary considerations is actually much more insidiously damaging than this, since it can lead to the postulation of psychological mechanisms that are, *a priori*, very unlikely to be true, and since, divorced from its function, we run the risk of misunderstanding even *how* the mechanism works.

The potential dangers of ignoring evolutionary considerations can be illustrated by the following series of studies examining performance on spatial memory tasks by nectar-feeding birds. Burke and Fulham (2003) showed that Regent Honeyeaters, an Australian nectar-feeding bird were much better able to learn to avoid a feeder that they had recently found nectar in (to win-shift) than they were able to learn to return to that location (win-stay). This is the same pattern found in other nectar feeding species (e.g., Kamil, 1978), and reflects the fact that, in the wild, a visited flower is depleted of nectar, and so avoiding such locations leads to efficient foraging. We could have postulated some memory/motivational mechanism that accounted for this behavior (indeed there were some general process ones already available in the psychology literature—Gaffan and Davies, 1981, 1982), but ecological considerations led us to test whether after a long delay (long enough for flowers in the wild to have replenished their nectar) this tendency might be reversed. This is what we found—at long delays, the birds actually more easily learnt to win-stay than to win-shift, despite all the birds in our study being captive born and reared, and so being unfamiliar with the natural replenishment rates of flowers. This finding demonstrates that the way the spatial memory mechanism underpinning returning to or avoiding rewarding locations *works* is intimately tied to it's adaptive function. We have subsequently followed this research up, probing the mechanism in more detail in a related omnivorous species (Noisy Miners), and determined that the win shift bias is expressed only when the reward is nectar, not when it is an invertebrate (as predicted from the spatio-temporal distribution of these two foods—Sulikowski and Burke, 2007), despite the tasks being identical in every way except for the nature of the reward. This effect is partly driven by the birds searching through arrays in different ways for nectar and invertebrates (Sulikowski and Burke, 2010a), partly by the fact that birds do not encode the spatial locations of invertebrate loaded feeders (Sulikowski and Burke, 2010b), instead moving systematically through the array, whereas they spontaneously encode the locations of nectar loaded feeders (Sulikowski and Burke, 2011). Careful analysis of foraging patterns also suggests that poor performance in win-stay conditions with a nectar reward is not a consequence of poor memory for rewarded locations, but probably reflects a selective inhibition of the win-stay behavior (Sulikowski and Burke, 2012).

None of these aspects of the way this particular mechanism (or mechanisms) works would even have been investigated without thinking about remembering spatial locations from an ecological perspective. The details are tightly tied to the foraging ecology of the birds in question, and operate differently depending on the reward being searched for. A straightforward, but not widely appreciated, implication of this is that it may well be *meaningless* to talk about a general spatial memory mechanism, in any species (including humans)—that psychological mechanisms can *only* be understood in their evolutionary/functional context. In the current example, *what* is remembered about rewarding spatial locations depends on the kind of food found there and the length of the “retention interval”—neither of these effects can be predicted by any general theory of memory (or even spatial or “working” memory), but both are predicted by the spatio-temporal distribution of the bird's food in the wild. There have been pushes to better incorporate mechanism in behavioral ecology (McNamara and Houston, 2009), and evolution and ecology in investigations of psychological mechanism (e.g., Kamil, 1988; Barkow et al., 1992; Buss et al., 1998; Shettleworth, 2010, etc.), but perhaps to ensure greater impact we should be emphasizing the fact that the two will frequently be intrinsically intertwined, and that one without the other won't just produce *incomplete* understanding, it may well produce complete *mis*understandings.

## The identification of evolutionary psychology with particular versions of it

Much of the explicit criticism of EP is clearly directed at just the most visible, and formally articulated versions of it, rather than being criticisms of an evolutionary *approach* to psychology in general. Indeed, some critics are explicit about this distinction (Buller, 2005), as discussed later. This is unfortunate because for those that don't closely follow the details of these debates, a criticism of particular versions of EP is taken as a criticism of the approach, and used as a justification for continuing to ignore evolution in psychology, and at least some of the things the critics have targeted are not arguments against the importance of an evolutionary approach.

While we have very good reasons to be grateful for the pioneering efforts of those that forged the field, it is probably time to explicitly acknowledge that not everyone taking an evolutionary approach to understanding psychology accepts all of the features that have been taken to be diagnostic of EP. The two main sticking points from outside the field seem to be the notion of massive modularity and that adaptations are “designed” to operate in the Pleistocene, but I argue below that there is also no *necessary* link between adopting an evolutionary approach and believing that the brain is a computational information processing device (even though all the major summaries of the perspective claim this as a central tenet of EP). Indeed, two papers in the current issue are arguing *for* an evolutionary approach to understanding psychology, but equate EP with a computational and modular approach (Barrett et al., 2014; Stotz, 2014).

Well-balanced and convincing arguments have been mounted from within the field defending the idea of modules for processing (to some extent) domain-specific information (e.g., Barrett and Kurzban, 2006), but much of the force of these arguments depends on the underlying assumption that the brain is an information processing device. In the absence of that assumption (discussed below), we can probably safely not commit ourselves to exactly *how* modular evolved mechanisms are likely to be, without in any way compromising our insistence that we need to understand the mechanism from an evolutionary perspective. The convoluted and interconnected way in which complex adaptations evolve means that we should probably expect some to be quite modular, and others to depend on components of pre-existing mechanisms, or even to co-evolve with other mechanisms. The immune system, for example can be thought of as a module (at least in terms of having a specific job, or set of related jobs, to do), but it “uses” the circulatory system to “get to” sites of infection. It is difficult to decide, therefore, whether the circulatory system should be counted as part of the immune system, and/or whether combating pathogens should be considered one of the jobs (part of the “input”) of the circulatory system. As this example hopefully illustrates, there is a real sense in which these decisions need not be made, since they don't help us to understand how any of the mechanisms in question work, or how they evolved. In the same way, I think that while it is critical to identify the function, and in some cases functions, a psychological mechanism performs, we need not worry about whether we would classify it as a module or not, and there is certainly no need to insist that solving specific (even mutually incompatible) adaptive problems will necessarily result in a massively modular brain. Indeed, incompatible functions will frequently lead to adaptive tradeoffs in the underlying mechanisms rather than to a divergence of underlying mechanisms. Elaborate sexual ornaments, for example (like peacock tails) are advantageous in terms of attracting mates, but are frequently constrained by natural selection, since they are energetic and survival impediments.

Similarly, although there is no formally articulated alternative, since all of the major summaries of the field subscribe to the information processing/computational metaphor (in common with the vast majority of cognitive psychologists), there is actually no logical link between such a metaphor of brain (or “mind”) function and an evolutionary approach. This metaphor is largely absent in behavioral ecology and ethology (including human ethology), for example, but those fields have made enormous contributions to our understanding of the evolution of behavior and behavioral mechanisms. In fact, it seems to me that thinking about brains from an evolutionary perspective actually undermines the information processing metaphor. Brains cannot be “for” processing information, because processing information has no fitness consequences. Gaining sensitivity to important environmental information can have fitness consequences, provided that information is appropriately acted upon, and brains are clearly involved in providing organisms with sensitivity to environmental information and in coordinating actions. My view is that the direct approach to cognition, like that espoused by Gibson (1979), which emphasizes dynamic, embedded organism-environment interactions, is a much more natural fit for an evolutionary approach, but like modularity, I think that meta-theoretical perspectives about the nature of cognition are not central to an evolutionary approach to Psychology, and so it is not appropriate or necessary to commit the field to any particular approach. This might have the additional benefit of attracting more biologists to study the evolution of psychological mechanisms. The impression I get from colleagues in biology is that many avoid psychological questions because they see things like the computational/representational approach as esoteric and unnecessary abstractions.

Consistent with the idea that we need not commit to either massive modularity or the information processing metaphor as characteristics of EP is the fact that fewer than 1% of papers published in the journals *Evolutionary Psychology* and *Evolution and Human Behavior* in 2013 (total 104—excluding the special issue of *EP*) in any way address, or are even informed by, these issues. Much more common (17%) is deriving hypotheses (or drawing conclusions) based on thinking about adaptive problems faced by our Pleistocene (or at least Hunter-Gatherer) ancestors, which is addressed in the next section. The vast majority of research in both journals (the other 80%) tests hypotheses derived from fundamental evolutionary principles.

## Just so story telling

Despite numerous attempts to explain exactly how evolutionary hypotheses are derived and tested (and occasionally rejected) in exactly the same way that other kinds of hypotheses in psychology are derived and tested, most recently by Confer et al. (2010), the idea that evolutionary hypotheses are somehow *intrinsically* untestable remains a pervasive view (Kurzban, 2010). Perhaps we might make more headway by more frequently acknowledging that evolutionary hypotheses are actually quite difficult to test (as have Confer et al., 2010, for example), and that psychological studies are but one of many lines of converging evidence that are helping to put together the pieces of the puzzle. It is probably a fair criticism of our field that we rely too heavily on uncovering signs of special design of human psychological mechanisms as evidence of their evolution, and too little on examining the mechanism across species (Vonk and Shackelford, 2013). Other fields that are interested in the evolution of behavioral mechanisms routinely make phylogenetic comparisons, to test hypotheses. Even where we are proposing the evolution of a uniquely human adaptation, cross-species comparisons are (ultimately) necessary to test that idea. Of course not every paper needs to include such comparisons (particularly since they are often logistically difficult), but we may gain more widespread acceptance (or at least less widespread resistance) by explicitly acknowledging that without such comparisons many conclusions need to remain tentative.

I am not here arguing that we need cross species comparisons to test *whether* a mechanism evolved—I think we need to be working toward a broad psychology in which that is an unquestioned assumption—but to test *how* it evolved—using knowledge of phylogeny and ecological selective forces. To illustrate this, consider Burke and Sulikowski's (2010) demonstration that backward tilted faces (simulating viewing from below) are judged as more masculine (or less feminine) and forward tilted faces (simulating viewing from above) are judged to be more feminine (or less masculine). Based on this, they concluded that the structural sexual dimorphism in human faces, with males having larger jaws and smaller eyes, and females having smaller jaws and larger eyes, may have evolved to accentuate, or just make structural, the different appearance of faces viewed from above (as females tend to be seen by males) and below (as males tend to be seen by females), since males and females also differ in average height. The data are *consistent* with this conclusion, but it is strengthened by the fact that all of the hominins (who are all bipedal) show marked sexual height dimorphism, and the same face dimorphism as humans, but that the other apes (who are not bipedal) do not show the same face shape dimorphism. Of course this alone is not sufficient to conclude that the face shape differences *are* a consequence of an evolved signal exploiting the height-based perspective difference, but it is corroborating. Further evidence is required to rule out other possibilities, but the point of this example is to highlight that necessary and sufficient evidence won't always come from Psychology, or from humans.

The impression I get from my colleagues is that part of the indelibility of the stain of “just-so story telling” is related to the idea that EP is fundamentally focused on explaining (all) human behavior in terms of what would have been useful to our Hunter-Gatherer ancestors. Although it is true that we have spent the vast majority of our time as a species living such a lifestyle, it is almost certainly not true that most of our cognitive adaptations are *for* a “stone age” world—almost all of them very likely to predate this epoch considerably, and some may be newer. For example, almost every adaptation for perceiving the world (accounting for something like half of the neurons in the brain), was in place long before this epoch, and the mechanisms underpinning lactose tolerance, and resistance to particular localized diseases (e.g., malaria, plague) appear to have arisen later (Schaffner and Sabeti, 2008). Given that most EP is not actually about testing hypotheses specific to this epoch, since most studies tests hypotheses derived from fundamental evolutionary principles, one way of overcoming this misconception might be to try to more widely publicize those kinds of studies.

## Motivated opposition

Despite a noticeable (if gradual) shift away from what Tooby and Cosmides (1992) originally identified as the Standard Social Science Model, there remain pockets of vigorous opposition to the evolutionary approach to psychology. The main problem with this opposition is not the logic of the arguments or the strength of the evidence they provide against EP—typically they are weak, or based on a misunderstanding (Kurzban, 2010)—it is the fact that any kind of formal opposition provides a rationale for mainstream psychologists to keep ignoring evolutionary approaches.

There are clear signs that this opposition is motivated, rather than an inevitable consequence of a careful analysis of the accumulated evidence. Naturally, claims for which there is insufficient evidence are a concern in any field, and it is appropriate therefore to invite as much scrutiny as possible, but EP is the kind of field that has long had to deal with criticism (unfortunately much of it based on the next two factors discussed), and so is probably less likely than most fields to make claims for which there is insufficient evidence. One sign that some critiques are motivated is that they draw substantially broader conclusions than are warranted by their data and/or analyses. For example, Buller (2000, 2005) claims to have no issue with EP as a *field of enquiry* (generally taking an evolutionary approach to psychological questions) but is rather scathing of EP as a *paradigm* (by which he seems to mean the research done by the most prominent practitioners). Despite having no (avowed) problems with EP as a field of enquiry, he makes the very broad claim that there is *no* good evidence for *any* of the psychological adaptations that have been proposed. It is not unreasonable to suppose that critiques that find flaws with *all* of the claims that have been made might not be weighing up evidence in a completely unbiased way.

To further illustrate the nature of the problem, I will focus on a more recent critic of EP research, Christine Harris, who has published two failures to replicate evolutionarily inspired studies reporting shifts in women's judgments across the menstrual cycle. The first called into question the well-known (and indeed well-established) fluctuations in attractiveness judgments (Harris, 2011) and the most recent “failed to replicate” a shift in voting preferences (Harris and Mickes, 2014). Clear and cogent responses to both of these have been published, by the original researchers (DeBruine et al., 2010; Gildersleeve et al., 2013; Durante et al., 2014), identifying flaws in logic and methodology, but it is the broader conclusions Harris tries to draw that I believe reveal an obvious bias. Having failed to replicate one particular study of shifts in women's preferences for masculinized faces across the menstrual cycle (and having failed to review the large body of corroborating evidence), Harris (2011) concludes that we should be questioning “much of the current work in evolutionary psychology,” especially those that identify “gender differences.” This, of course, is not in any way warranted by the data, suggesting an obvious agenda. Similarly, despite a provocative (and politically charged) title—“Women can keep the vote: No evidence that hormonal changes during the menstrual cycle impact political and religious beliefs”—Harris and Mickes (2014) actually *did* replicate the interaction between menstrual cycle phase and relationship status on voting intentions—the most interesting aspect of the original study that they claim to have failed to replicate. Rather than attempting to get to the bottom of such an intriguing effect, their final conclusion is that their data add to a “growing number of failures to replicate several menstrual cycle effects on preferences” (they cite two), and essentially insinuate that the previous (very numerous) reports of positive effects of menstrual cycle phase on preferences are a consequence of “flexible” data analysis and fertility status classifications (for which there is no evidence).

It is difficult to be sure, but the tone of Harris' opposition to the evidence of menstrual cycle shifts in judgments suggests that it is based on the idea that such conclusions are somehow sexist—that they suggest that women's decisions are in some sense “at the mercy of their hormones.” But I take the main message of this research to be that we are *all*, in some sense at least, at the mercy of our hormones (and not just gonadal ones), as they influence our decisions in evolutionarily adaptive ways. The preponderance of studies examining fluctuations across the menstrual cycle is almost certainly a simple consequence of the natural pseudo-experiment afforded by monthly variations in hormone balances. To look for the same effects in men, hormone levels need to be actually measured or manipulated, which makes such studies less common, but there is good evidence of strong effects of hormones on male behavior too (e.g., Mazur and Booth, 1998).

Of more concern than the opposition of any individual researcher (or group) is what quite obvious biases in published papers suggest about the broad attitudes of the field. It is worth wondering, for example, whether the “failed” replication of the Durante et al. (2013) paper would have ever been published in the absence of a broad (if potentially subtle) bias against evolutionary explanations (and/or those proposing sex differences based on something other than socialization differences) in mainstream psychology. Not only did the paper actually *not* fail to replicate the primary finding, it misrepresented the original authors' rationale (in a way that is consistent with well-known misunderstandings about evolutionary approaches being inherently sexist), was published despite at least one of the reviewers having sufficiently serious misgivings that they re-analyzed the data and found even more *consistencies* between the two studies, and was allowed to go to print with a title that clearly suggested that the original research *was* sexist, and with a conclusion that smears the entire body of literature examining shifts in preferences across the menstrual cycle. These are excesses that are typically not permitted. That they were permitted in a high-impact, mainstream psychology journal suggests the influence of a pervasive bias.

## Theoretical inertia and misguided skepticism

In general, in Science, skepticism is an invaluable tool, since it minimizes the risk of drawing conclusions on too little data, and especially of discarding existing theories without sufficient justification. But skepticism is frequently asymmetrical, with new approaches being more intensely scrutinized than old approaches. This is justified if the old approach is built on solid foundations, and has had much explanatory success, but there are good reasons for questioning whether this is true of many theories in psychology, especially since evolution was not one of the basic principles upon which they were built. I think that this asymmetrical skepticism might be at the heart of at least some of the bias against evolutionary approaches in mainstream psychology, even in the absence of any obviously motivated opposition.

The impression I have of the attitude of many of my colleagues is that there is no real *need* to adopt an evolutionary approach because psychology is doing fine without one, and this is associated with a reluctance to accept even *demonstrations* of the importance of an evolutionary perspective, with skeptics arguing that existing mechanisms (typically general process ones) are capable of explaining the results, and so there is just no need to propose “new” mechanisms. No doubt everyone who adopts an evolutionary approach in psychology has had to argue against these kinds of perspectives in their own sub-field, but in order to draw attention to the pervasiveness of the problem, I'd like to use an example of a general process mechanism that is accepted even by many evolutionarily oriented researchers (e.g., Shettleworth, 2010; Cosmides and Tooby, 2013)—the idea that there are general associative learning mechanisms.

The widespread acceptance of this view is an example of skepticism being directed only at new evidence, not at the evidence that underpins the traditional perspective. In fact, I think it is perfectly reasonable to claim not only that there is no good evidence that associative learning mechanisms are phylogenetically widespread (let alone evolutionarily conserved), but to question the very idea that *any* associative learning *mechanisms* have been established, at all. I understand that this claim seems extreme, but it is important to keep in mind that when we refer to classical (or Pavlovian) conditioning or to instrumental (or operant) conditioning, we are referring to learning *situations*—experimental paradigms that have been extensively used to study learning. What is actually learnt in these paradigms is very much a matter of ongoing debate (e.g., Gallistel, 1995; Gallistel and Gibbon, 2000), and it clearly depends on what is being learnt about, and which species is doing the learning (as famously demonstrated by Garcia and Koelling, 1966; Shettleworth, 1973; Timberlake, 2001). It is true that using a neutral stimulus to predict the arrival of a biologically (or at least behaviorally) significant stimulus (as in a Pavlovian conditioning experiment) leads to the production of anticipatory/preparatory behaviors in response to the previously neutral stimulus in a wide range of species, but this is no more evidence of a common *mechanism* in those species than the observation that a wide range of species can move from point A to point B is evidence of a common locomotion mechanism. The trouble here is that psychologists, as they rather too frequently do, have conflated a mechanism (*how* something works) with a functional category of behavior (what something *does*). There is actually no good evidence of universality of mechanism—indeed, an argument could be mounted that there is not a single species in which we understand *how* behavior is adjusted to exploit these simple environmental contingencies, short of the not especially helpful suggestion that the environmental association between the stimuli has somehow been “copied” inside the organism.

I have chosen this likely controversial example to try to illustrate that even ideas that seem so well established that they are essentially beyond question in psychology owe at least some of their power and influence to a long history of investigation, but that those factors are unrelated to the likelihood of the ideas being true. Given that almost all of the longest-established ideas in Psychology pre-date an evolutionary approach, we should expect a reluctance to accept the need to factor evolution in. Maybe the only way to overcome this resistance is to start using an evolutionary approach to dismantle some of those ideas, not by just suggesting that the standard social science model is an inappropriate one, given what we know about how mechanisms actually evolve, but by actively targeting particular (maybe especially popular) theories that cannot be easily accommodated within an evolutionary framework.

## Poor understanding of modern evolutionary principles

I think the most fundamental problem in the more widespread acceptance of an evolutionary approach in psychology is the fact that very few psychology researchers or practitioners actually understand evolution, a problem that is considerably compounded by the fact that they are typically completely unaware of this. This is likely to be a consequence of the fact that most psychology degrees do not contain a good grounding in evolutionary theory. I teach at a well-rated university in a Psychology School that was one of only a handful in the country (Australia) to receive a 5 star ranking in the latest national quality assessment exercise, and I recently asked an advanced undergraduate class (in their 4th year) if they could describe the difference between natural and sexual selection. Only five (out of 113) of the students confidently knew the difference, despite evolutionary approaches being one of the topics (briefly) covered in the class. My students probably get exposed to more evolutionarily oriented psychology than most (certainly in Australia), but they do not, as is typical, do a class on evolution, and so they can't really be expected to have a proper appreciation of the insights such an understanding provides. An ability to even understand the importance of an evolutionary perspective in psychology depends, I think, on genuinely understanding how evolution works, and so we need to do what we can to pass on this *fundamental* knowledge if we hope to make evolution central in psychology. If our students (and I know this is also true of almost all of my colleagues) don't know the difference between sexual and natural selection, then they almost certainly don't know about Hamilton's rule and inclusive fitness, Trivers' Parental Investment Theory, condition-dependent strategies, honest signaling of mate quality, and a host of other concepts that are central to understanding the evolution of behavioral mechanisms in general. Given this, it is not surprising that they don't fully appreciate the power and importance of an evolutionary approach to psychology.

A clear illustration of this problem can be seen in many existing theories and debates in Psychology, perhaps most tellingly, even those purporting to be “evolutionary.” For example, Ekman's (1992, 1997) well-known theory of universal emotion recognition and production is taken to be an evolutionary theory because there is cross-cultural consistency in the way in which the “basic” expressions are labeled. But the fundamental premise of the theory—that one's emotions erupt uncontrollably on the face, thus communicating them—is at odds with a modern understanding of the evolution of communicative signaling, in which the costs and benefits to the signaler and the receiver need to be weighed up, and in which a great deal of “communication” functions to “manipulate” other individuals (Krebs and Dawkins, 1984). A properly informed evolutionary perspective encourages us to ask how the expressions that we display increase our fitness, and how detecting and responding to them affects the fitness of the receivers.

Similarly, a great deal of debate in the face (identity) perception literature has focused on whether the Fusiform Face Area (FFA) only “processes” faces, or whether it is actually a part of the brain “for” perceiving any object that is habitually categorized at the subordinate level and with which we have substantial experience, and therefore expertise (e.g., Kanwisher et al., 1997; Gauthier et al., 1999). This debate has been widely construed as one between those who believe that there is an “evolved” “special” face area and those who hold that the apparent specialness is a consequence of expertise and the unusual nature of the stimuli being perceived. If the protagonists in this debate had a better grounding in the nature of evolved adaptations, they would not be using evidence that experience makes a difference to how some objects are “processed” to decide whether FFA is an evolved face perception area, since such effects are essentially orthogonal to whether the area originally evolved “*to*” perceive faces (Barrett, 2012; Burke and Sulikowski, 2013). Indeed, the fact that people can learn to use FFA to discriminate between “greebles” (artificial stimuli that differ in configural ways like faces) tells us as much about the evolved function of FFA as the fact that people can learn to ride bicycles tells us about the evolved function of legs.

## What to do?

To some extent EP is a victim of its own success. I think we all agree that stand-alone degree programs, and specialist conferences and journals are an important part of the field developing an identity and progressing without having to have protracted (and pointless) debates with those opposed to our approach, but they also have a tendency to isolate EP researchers (and maybe especially the new generation who are coming through the programs) from the core evolutionary biology and behavioral ecology that originally formed the inspiration for our discipline, and also from mainstream psychology. This isolation/protection has the potential to reduce the “selection pressure” on the field, and so to enable the proliferation of approaches that fall under the EP umbrella that are less rigorous than they would otherwise be. We would be wise to guard against this, to avoid providing opponents with genuine ammunition. Of course, it is almost inevitable that every area will produce some poor research, but given that EP faces motivated opposition in a way that most other sub-disciplines of psychology don't, and depends on a core of knowledge that most of our colleagues don't have, we need to be especially careful to ensure that our output is as rigorous and well-informed as it can possibly be. It might also be helpful to be conscious of the nature of the opposition our findings may face, and the ways in which they may be misunderstood, and to preemptively allay them in our published papers, and especially in our dealings with the media (when this is possible).

In addition to courses on EP itself (ideally with comparative psychology integrated into them), I think it is important that all psychology students learn basic evolutionary biology and behavioral ecology (and maybe physical anthropology where such classes still exist)—completely independently of psychology. This comprises much of the core knowledge they need to approach psychology from an evolutionary perspective, both in terms of the actual content of such classes, but also in the mere fact of being exposed to complex adapted mechanisms in a wide range of species, giving them the appropriate perspective on human behavioral mechanisms. I suspect that without producing a generation of psychology students who properly understand evolution, we will always be fighting a losing battle to have evolutionary approaches integrated into mainstream psychology. Even if we could, overnight, instill a burning *desire* in all psychologists to approach their research from an evolutionary perspective, this would likely hinder more than help our field because they would be *unable* to do research that is properly informed by an understanding of evolution.

Although I think it is important to publish our *findings* in mainstream psychology journals (arduous though this task can be), I think it might actually be a good idea to *stop* trying to explain what EP *is* to those outside the field. So far that seems to have served mostly to focus opposition, and as I have argued here, some of that opposition might be at least partially justified. As a brief survey of the kinds of papers being published in the field shows, the summaries that have been produced don't really reflect the majority of the research being conducted, anyway. I wonder if a more effective strategy might be to instead target mainstream (ideally high impact) outlets for findings that either would never have been investigated without an evolutionary approach, or of phenomena that make no sense except in light of evolution. EP is also a very media-friendly discipline (something that I suspect makes us more of a target from our mainstream colleagues than we might otherwise be). Ideally, we would be able to use that interest in a more strategic way than we currently do, again, by making more widely known studies in which aspects of human psychology only make sense in light of well-established, general evolutionary principles—the kinds of findings that don't depend on any untested assumptions about our recent ancestors, or the structure and nature of our cognitive mechanisms, but rather are straightforward, essentially irrefutable corollaries of fundamental evolutionary principles. A good example of such a finding is the MHC-dependent odor preferences discovered by Wedekind et al. (1995). These are the kinds of findings that I believe are most likely to convince skeptics of the value of our approach, and which could lay the foundations of a psychology that genuinely integrates evolution.

### Conflict of interest statement

The author declares that the research was conducted in the absence of any commercial or financial relationships that could be construed as a potential conflict of interest.
